# Supplementary material for: Slow growing behavior in African trypanosomes during adipose tissue colonization
Source: Nat Commun. 2022 Dec 8;13:7548. doi: 10.1038/s41467-022-34622-w (PMC9732351; doi:10.1038/s41467-022-34622-w)
Supplement: Supplementary file 1 — Supplementary Information [file 41467_2022_34622_MOESM1_ESM.pdf]

Supplementary Information for

Slow growing behavior in African trypanosomes during adipose tissue colonization

Sandra Trindade, Mariana De Niz, Mariana Costa-Sequeira, Tiago Bizarra-Rebelo, Fábio Bento, Mario Dejung, Marta Valido Narciso, Lara Lopez-Escobar, João Ferreira, Falk Butter, Frédéric Bringaud, Erida Gjini & Luisa M. Figueiredo.

Luisa M. Figueiredo, Erida Gjini
Email: lmf@medicina.ulisboa.pt and erida.gjini@tecnico.ulisboa.pt

This PDF file includes:

Supplementary Figures 1 to 9
Supplementary Tables 1 to 9
Supplementary text
SI References

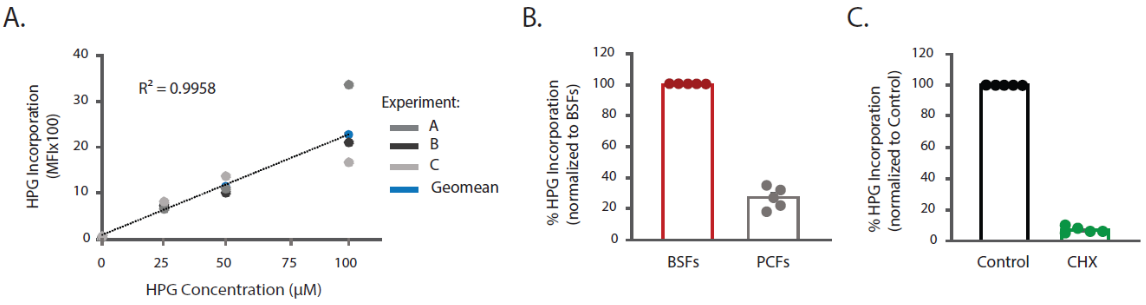

Supplementary Figure 1| Experimental Procedure Optimization and Validation. A, Mean Fluorescence Intensity of HPG incorporation by culture monomorphic parasites. Three increasing concentrations were used to test for saturating conditions. Basal levels of HPG MFI were assessed with the 0µM HPG condition (n=3 independent experiments per condition). **B,** Percentage of HPG internalized by PCFs (grey circles, bar) normalized to the percentage of internalization of the BSFs (red circles, bar) (n=5 independent experiments). **C,** Percentage of HPG incorporation by culture monomorphic Lister 427 parasites exposed to 100µg/mL of cycloheximide (CHX) for 20 minutes (green circles, bar) normalized to the percentage of internalization of cultured parasites without protein synthesis inhibition (black circles, bar) (n=5 independent experiments). Error bars represent the standard error of the mean.

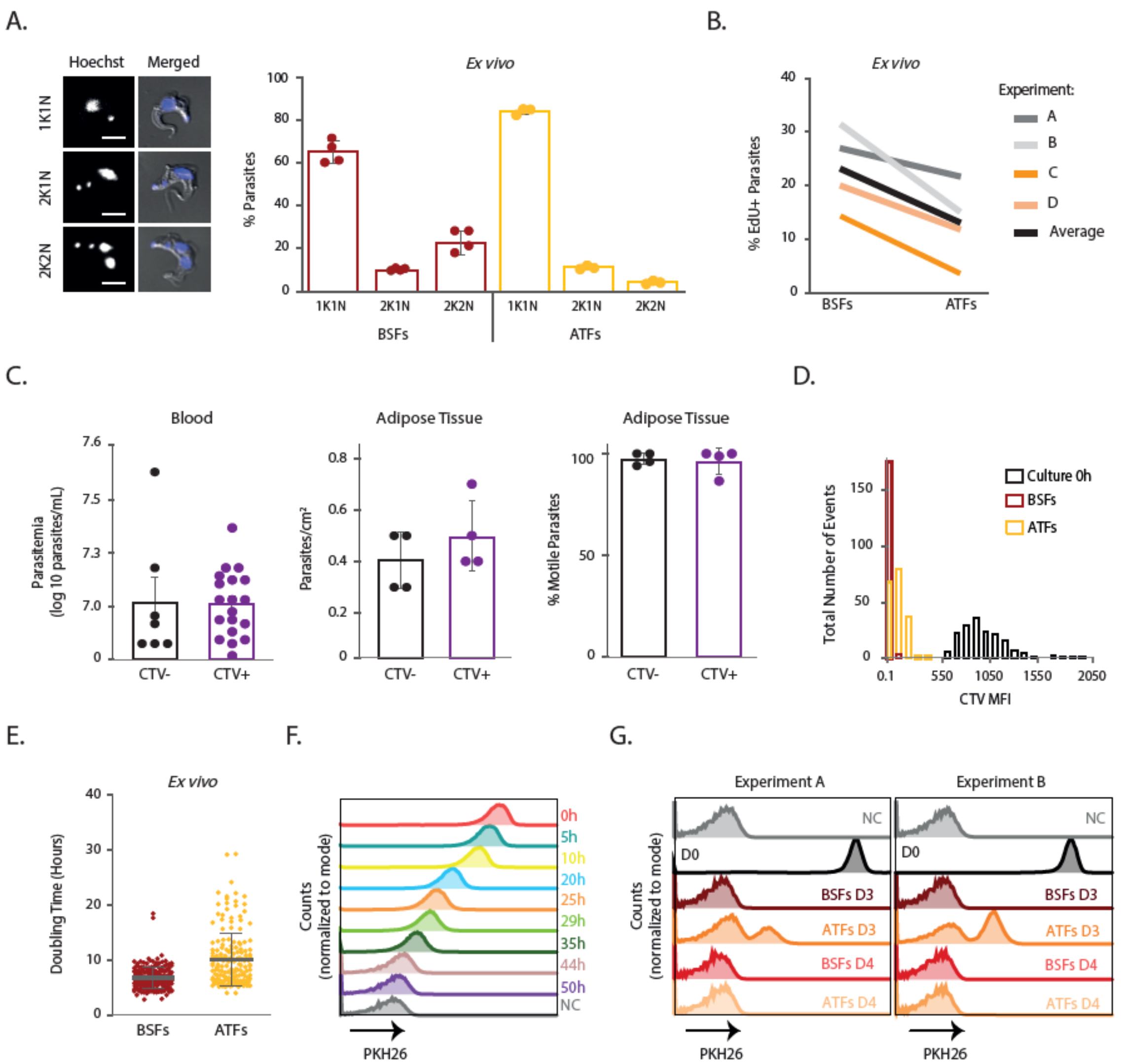

Supplementary Figure 2| Analysis of cell cycle profile and cell proliferation. A, *Ex vivo* cell-cycle analysis assayed by the number of Kinetoplasts (K) and Nuclei (N) in BSFs and ATFs Lister 427 parasites isolated from mice infected for 5 days (n=4 independent experiments). Representative images of the three parasites analyzed *ex vivo* (Hoechst staining, white in the Hoechst column and blue in the Merged column; scale bar, 5µm) and histogram of the percentage of BSFs (red circles, bars) and ATFs (yellow circles, bars) in each configuration. More than 600 cells per condition. **B,** *Ex vivo* microscopy analysis of BSFs and ATFs isolated from a pool of 2 mice infected for 5 days and labeled with EdU (n=4 independent experiments). Reaction norm represents the percentage of EdU positive parasites. More than 300 cells per condition. **C,** Parasitemia, parasite density (parasites/cm^2) and percentage of motile parasites in gonadal adipose tissue of mice infected for 2 days with either non-labeled (CTV-) (black circles, bars) or CTV labelled (CTV+) (purple circles, bars) Lister 427 parasites (Parasitemia: n=7 CTV- and n=19 CTV+ pooled from the 4 independent experiments, parasites/cm^2, % motile parasites: n=4 independent experiments). **D,** Distribution of the CTV Mean Fluorescence Intensity of culture parasites, BSFs and ATFs assessed by intravital microscopy (n=202 culture parasites, n=183 BSFs and n=199 ATFs pooled from the 4 independent experiments). First bin of 50, remaining bins of 100. **E,** Doubling time estimated by *ex vivo* microscopy analysis for BSFs (red circles) and ATFs (yellow circles) isolated from mice infected with cultured Lister 427 parasites labeled with (n=209 BSFs and n=183 ATFs pooled from the 4 independent experiments). Grey lines represent the average values. **F,** Representative Flow cytometry profiles of cultured Lister 427 parasites non-

labeled (NC) and labeled with PKH26 at different time points post-labeling (n=3 independent experiments). **G,** Flow cytometry profiles of non-labeled cultured Lister 427 parasites, cultured parasites labeled with PKH26 and BSFs and ATFs isolated from mice infected for 3 and 4 days (n=3 independent experiments). Error bars represent the average and the standard error of the mean (C: parasitemia) and standard deviation (A, C: parasites/cm^2, % motile parasites, E). NC: Negative Control, D0: Day0, D3: Day3, D4: Day4.

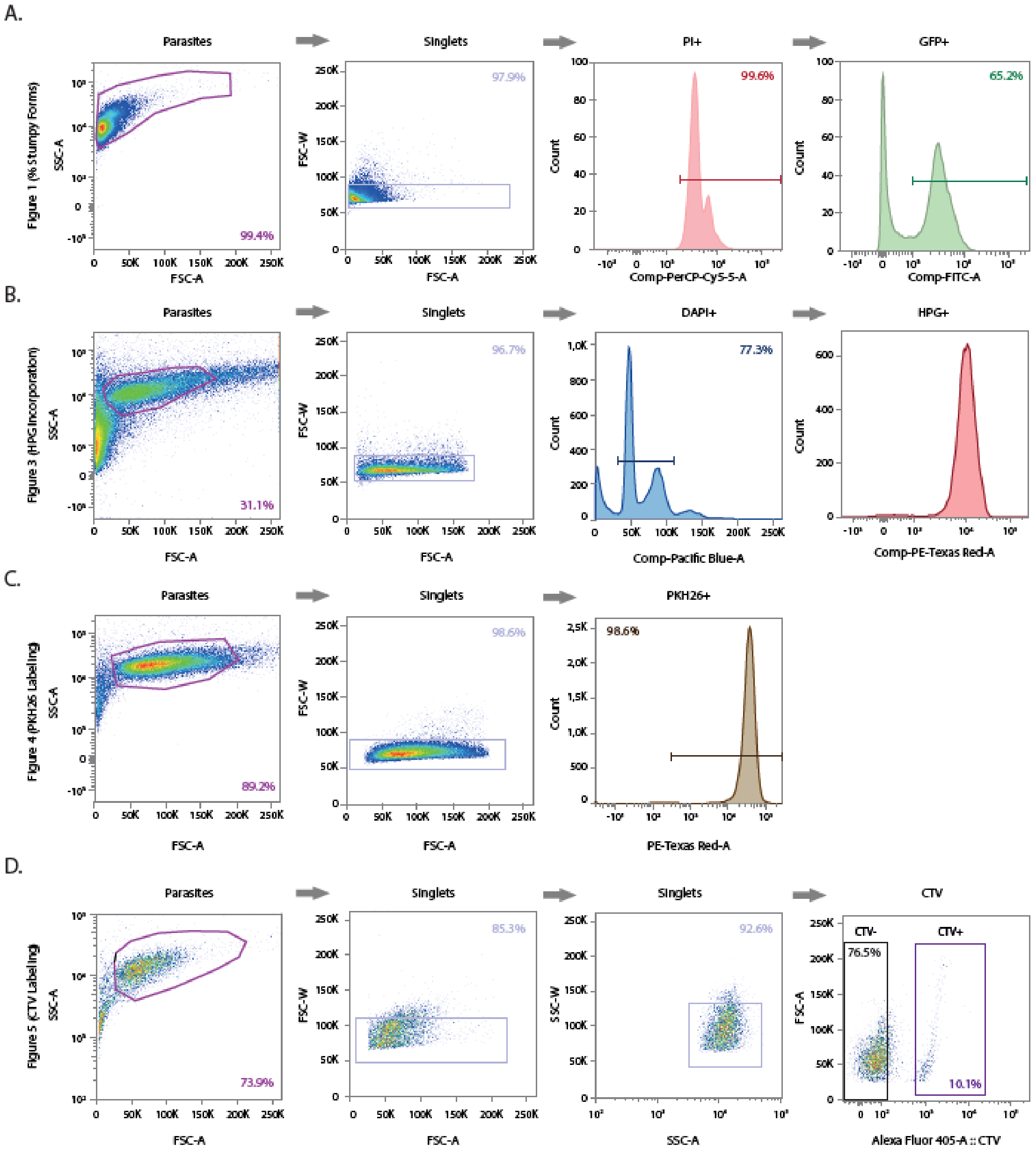

Supplementary Figure 3| Flow cytometry gating strategy. Representative flow cytometry profiles of the gating strategy used to: **A,** determine the percentage of stumpy forms represented in figure 1 (ATFs isolated from a mouse infected for 7 days with a pleomorphic *T. brucei GFP::PAD1utr* stumpy reporter cell-line). **B,** determine the percentage of HPG incorporation represented in figure 3 (ATFs isolated from a pool of 3 mice infected for 5 days with Lister 427 parasites). **C,** define the existence of PKH26 labeled parasites represented in figure 4 (cultured Lister 427 parasites labeled with PKH26). **D,** to sort CTV negative (CTV-) and CTV positive (CTV+) parasites represented in figure 5 (ATFs isolated from a mouse infected for 3 days with Lister 427 parasites). Grey arrows represent the direction of the gating strategy. Percentages represent the percentage of gated cells.

Supplementary Table 1. Relative parasite density and total parasite load in blood and adipose tissue.

Day Post Infection	Mice	Parasite Density (Parasites/mg)		Total Organ Weight (mg)		Parasite Load (Parasites/Organ)	
		Blood	Adipose Tissue	Blood″	Adipose Tissue	Blood	Adipose Tissue
4	1	8	30*	1404	398	11339	11940
	2	815	14290	1404	397	1144509	5673241
	3	7	30*	1404	265	9931	7950
5	1	25227	20000	1404	460	35418272	9200076
	2	52566	4364	1404	499	73802727	2177862
	3	¥	4114	1404	383	¥	1575532
6	1	72056	16631	1404	531	101166945	8831086
	2	123882	21604	1404	372	173930539	8036750
	3	138960	29803	1404	381	195100171	11354974
7	1	9	54935	1404	284	12004	15601569
	2	26514	12695	1404	325	37225486	4125932
	3	7	37625	1404	386	10186	14523423
8	1	2	17363	1404	321	3086	5573617
	2	¥	312	1404	449	¥	139880
	3	1	743	1404	297	1890	220524
9	1	18	70	1404	313	24926	21835
	2	¥	107	1404	332	¥	35441
	3	4	63	1404	269	5791	16882
10	1	59	¥	1404	226	82332	¥
	2	31	380	1404	260	42904	98841
	3	19	41	1404	163	26383	6649
11	1	168	209	1404	126	235973	26277
	2	435	781	1404	251	611392	195943
	3	311	4742	1404	264	436034	1252007
13	1	8821	63065	1404	363	12384062	22892612
	2	5029	14850	1404	244	7060073	3623350
14	1	1009	8462	1404	116	1416081	981638
	2	104	304	1404	104	145869	31620
15	1	6269	22250	1404	93	8801597	2069264
	2	11661	41196	1404	93	16372350	3831246
	3	4650	12179	1404	136	6528724	1656365
16	1	10300	13870	1404	155	14461572	2149925
	2	3836	12112	1404	95	5385861	1150620
	3	2022	¥	1404	104	2839301	¥
20	1	1703	2482	1404	79	2391538	196048
	2	4009	7132	1404	74	5628083	527790
	3	4079	10243	1404	78	5726882	798933
21	1	10342	9690	1404	78	14520792	755844
	2	41440	114136	1404	220	58181662	25109959
	3	298	46239	1404	162	418498	7490706
	4	11529	71568	1404	168	16186946	12023501
26	1	7323	19940	1404	86	10281503	1714803
	2	1883	17040	1404	127	2644354	2164052
	3	10499	21935	1404	97	14740996	2127737
27	1	3131	630	1404	118	4395913	74370
	2	2152	4160	1404	141	3021611	586583
28	1	75	61417	1404	89	104982	5466106
	2	10605	55771	1404	100	14889843	5577137
	3	25052	¥	1404	122	35172473	¥

* Atributed value (half the detection limit)

″ Mice have 58.5 ml of blood per kg of bodyweight. Mice at this age weight on average 24mg. Blood density is 1000mg/mL.

¥ Deteriorated samples

Supplementary Table 2. Fixed parameters of mathematical models.

Parameter	Interpretation	Value	Units
p_o	Initial Density of Parasites in Blood	1	Cells/mg
I_o	Initial Level of VSG-specific Response	1	-
n	Number of Antigenic Waves Over 28 Days	5	-
δ	Mortality rate of stumpy cells	0.5	Per Day
τ	Initial Delay for Immune Response Activation	4	Days

Supplementary Table 3. Comparison of three mathematical models using DIC and LRT scores.

Model	Parameter Difference	Biological Assumption	DIC*	LRT*1
1	$r_b = r_f, K_b = K_f$	No Differences Between Blood and Adipose Tissue	173.42	67.65 (M1 vs M2)
				Favors More Complex Model 2
2	$r_b \neq r_f$	**Growth Rate** Varies Between Blood and Adipose Tissue	86.1	
				0.46 (M2 vs M3)
3	$r_b \neq r_f, K_b \neq K_f$	**Growth Rate** and **Differentition** Differ Simultaneously	85.13	*Favors Simpler Model 2*

* Deviance Information Criterion

*1 Likelihood Ratio Test

Supplementary Table 4. Gene Ontology enrichment of differentially expressed protein groups between blood and adipose tissue parasites.

Biological Process	Protein ID	Annotation	neglog10.pval
**Upregulated Genes in ATFs**			
**Purine Ribonucleoside Salvage (5/12) GO:0006166**	Tb927.10.1390	Hypoxanthine-guanine phosphoribosyltransferase, putative	6.7
	Tb927.10.1400; Tb927.10.1470	Hypoxanthine-guanine phosphoribosyltransferase	
	Tb927.2.6150; Tb927.9.7470	Adenosine transporter 2	
	Tb927.3.2960	Inosine-adenosine-guanosine-nucleosidehydrolase	
	Tb927.7.1780	Adenine phosphoribosyltransferase, putative	
**Metabolic Process (8/93) GO:0008152**	Tb927.10.12700	Pyruvate dehydrogenase E1 alpha subunit, putative	5.9
	Tb927.10.3210	Delta-1-pyrroline-5-carboxylate dehydrogenase, putative	
	Tb927.11.11680	2-oxoglutarate dehydrogenase E2 component, putative	
	Tb927.11.1450	2-oxoglutarate dehydrogenase E1 component, putative	
	Tb927.11.2690	Succinyl-coA:3-ketoacid-coenzyme A transferase, mitochondrial precursor, putative	
	Tb927.11.9980	2-oxoglutarate dehydrogenase E1 component, putative	
	Tb927.3.1790	Pyruvate dehydrogenase E1 beta subunit, putative	
	Tb927.4.2240	Aminotransferase class IV, putative	
**Oxidation-reduction Process (8/187) GO:0055114**	Tb927.5.300; Tb927.5.300b; Tb11.v5.0215	Thymine-7-hydroxylase, putative; 2-oxoglutarate (2OG) and Fe(II)-dependent oxygenase superfamily protein, putative; Iron/ascorbate oxidoreductase family protei	4.0
	Tb927.10.3210	Delta-1-pyrroline-5-carboxylate dehydrogenase, putative	
	Tb927.10.9360	L-2-hydroxyglutarate dehydrogenase, mitochondrial, putative	
	Tb927.11.1450	2-oxoglutarate dehydrogenase E1 component, putative	
	Tb927.11.9980	2-oxoglutarate dehydrogenase E1 component, putative	
	Tb927.5.3350	Iron superoxide dismutase A, mitochondrial	
	Tb927.7.7500	Thymine-7-hydroxylase, putative	
	Tb927.9.5900	Glutamate dehydrogenase	
**Downregulated Genes in ATFs**			
**Translation (49/208) GO:0006412**	Tb927.4.1100; Tb927.11.680; Tb11.0880	Ribosomal protein L21E (60S), putative; chrXI additional, unordered contigs	44.9
	Tb927.7.5020; Tb927.7.5000; Tb11.v5.0182; Tb11.v5.0181	60S ribosomal protein L19, putative	
	Tb927.10.5340; Tb927.10.5330; Tb11.v5.1060; Tb11.v5.0243	40S ribosomal protein S18, putative	
	Tb927.11.3600; Tb927.11.3590; Tb11.v5.1059	40S ribosomal protein S4, putative	
	Tb927.1.3180	40S ribosomal protein S11, putative	
	Tb927.10.10010	mRNA turnover protein 4 homolog, putative	
	Tb927.10.1090; Tb927.10.1080	40S ribosomal protein S23, putative	
	Tb927.10.1100; Tb927.6.4690	60S ribosomal protein L9, putative	
	Tb927.10.11390	60S ribosomal protein L6, putative	
	Tb927.9.6070; Tb927.10.11540	40S ribosomal protein S3, putative	
	Tb927.11.9730; Tb927.10.12680	60S ribosomal protein L34, putative	
	Tb927.11.9710; Tb927.10.13500	60S ribosomal protein L10a, putative; 60S ribosomal protein L10a	
	Tb927.11.4820; Tb927.10.14580	60S ribosomal protein L17, putative	
	Tb927.10.14710; Tb927.10.14600	40S ribosomal protein S2, putative	
	Tb927.2.5910; Tb927.10.15120	40S ribosomal protein S13, putative	
	Tb927.9.15210; Tb927.10.1590	Ribosomal protein L36, putative	
	Tb927.10.6370; Tb927.10.220	60S ribosomal proteins L37, putative; 60S ribosomal protein L37a, putative	
	Tb927.10.3280	60S ribosomal protein L38, putative	
	Tb927.10.3380; Tb927.10.3370	60S acidic ribosomal protein P2, putative	
	Tb927.11.14130; Tb927.10.3840	60S Ribosomal protein L18, putative; 60S ribosomal protein L18a, putative	
	Tb927.10.4110; Tb927.10.4120	60S ribosomal protein L30	
	Tb927.10.5030;Tb927.7.3680	Ubiquitin/ribosomal protein S27a, putative	
	Tb927.10.5370; Tb927.10.5360	40S ribosomal protein S10, putative	
	Tb927.10.5480; Tb927.10.5460	60S ribosomal protein L24, putative	
	Tb927.8.1110; Tb927.10.5610	40S ribosomal protein S9, putative	
	Tb927.10.7340; Tb927.10.7330	40S ribosomal protein S24E, putative	
	Tb927.11.4300; Tb927.10.9880	60S ribosomal protein L18, putative	
	Tb927.11.10030; Tb927.11.10025	60S ribosomal protein L29, putative	
	Tb927.11.10910; Tb927.11.10790	40S ribosomal protein SA, putative	
	Tb927.11.11725; Tb927.11.11475	Ribosomal protein S14p/S29e	
	Tb927.11.16280; Tb927.5.1110	60S ribosomal protein L8, putative	
	Tb927.9.9210; Tb927.11.3000	60S ribosomal protein L37, putative	
	Tb927.2.6090; Tb927.11.3230	60S ribosomal protein L44	
	Tb927.3.4360; Tb927.11.6140	40S ribosomal protein S15A, putative	
	Tb927.11.6200; Tb927.11.6180	60S ribosomal protein L28, putative	
	Tb927.11.6300	40S ribosomal protein S5, putative	
	Tb927.11.7685; Tb927.11.7675	Ribosomal protein S10p/S20	
	Tb927.11.8200	Ribosomal protein S26, putative	
	Tb927.3.3320; Tb927.3.3310	60S ribosomal protein L13, putative	
	Tb927.3.5050	60S ribosomal protein L4	
	Tb927.4.1800; Tb927.4.1790	Ribosomal protein L3, mitochondrial, putative; Ribosomal protein L3, putative	
	Tb927.7.2370; Tb927.7.2340	40S ribosomal protein S15, putative	
	Tb927.7.5180; Tb927.7.5170	60S ribosomal protein L23a, putative; 60S ribosomal protein L25	
	Tb927.9.11410; Tb927.9.11380	60S ribosomal protein L23, putative	
	Tb927.9.11470; Tb927.9.11490	60S ribosomal protein L29	
	Tb927.9.15170; Tb927.9.15110	60S ribosomal protein L5, putative	
	Tb927.9.5690	60S acidic ribosomal protein, putative	
	Tb927.9.7620; Tb927.9.7590	60S ribosomal protein L11, putative	
	Tb927.9.8420; Tb927.9.8070	QM-like protein	
**Ribosome Biogenesis (7/32) GO:0042254**	Tb927.10.10010	mRNA turnover protein 4 homolog, putative	5.4
	Tb927.10.1100; Tb927.6.4690	60S ribosomal protein L9, putative	
	Tb927.9.6070; Tb927.10.11540	40S ribosomal protein S3, putative	
	Tb927.10.5030; Tb927.7.3680	Ubiquitin/ribosomal protein S27a, putative	
	Tb927.11.3120	Nucleolar GTP-binding protein 1	
	Tb927.7.5180; Tb927.7.5170	60S ribosomal protein L23a, putative; 60S ribosomal protein L25	
	Tb927.7.5020; Tb927.7.5000; Tb11.v5.0182; Tb11.v5.0181	60S ribosomal protein L19, putative	

Supplementary Information Text
Supplementary Model Information.

The mathematical model for trypanosome infection dynamics

We build a deterministic ordinary differential equation model for densities of trypanosome parasites in different tissues. The model is largely based on a previous formulation (1), but now expanded for more in-host compartments (Fig. S4).

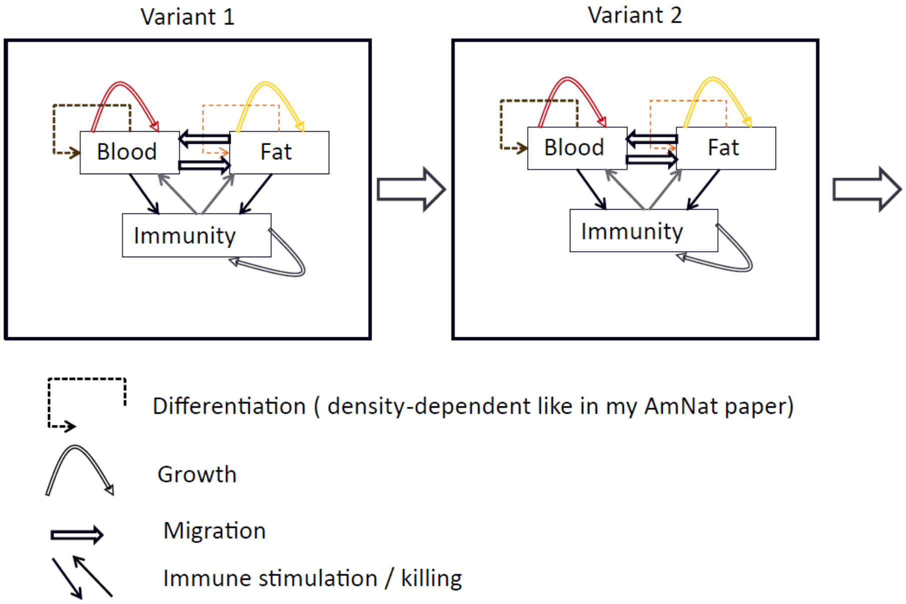

Supplementary Figure 4| Schematic diagram of the infection model dynamics (Eqs. 1-5). The infection processes modeled are based on (1) but here we simplify the equation for the immune response using a Holling-type functional response for antigen stimulation, and add compartment-specific infection variables connected via migration. Antigenic variation is modeled at the level of waves, allowing for up to 5 blocks of variants to appear over 28 days, and assuming a fixed block-to-block switch rate. All parameters are assumed constant throughout infection and symmetric across VSG.

Antigenic variation is modelled at the level of antigenic waves, capturing global peak dynamics. For all parasite antigenic waves emerging after the first wave, i = 2; ::n we describe the temporal dynamics of slender (v) and stumpy cells (m) in the blood and fat compartments (subscripts b and f) through the following set of differential equations:

$$\frac{dv_i^b}{dt} = r_b\left(1-\frac{V^b+M^b}{K_b}\right)\left((1-s)v_i^b+sv_{i-1}^b\right)-dv_i^bE_iI_{on}(t)-\mu v_i^b+\mu v_i^f \quad (1)$$

$$\frac{dm_i^b}{dt} = r_b\frac{V^b+M^b}{K_b}\left((1-s)v_i^b+sv_{i-1}^b\right)-d'm_i^bE_iI_{on}(t)-\mu m_i^b+\mu m_i^f-\delta m_i^b \quad (2)$$

$$\frac{dv_i^f}{dt} = r_fv_i^b\left(1-\frac{V^f+M^f}{K_f}\right)-dv_i^fE_iI_{on}(t)-\mu v_i^f+\mu v_i^b \quad (3)$$

$$\frac{dm_i^f}{dt} = r_fv_i^f\frac{V^f+M^f}{K_f}-d'm_i^fE_iI_{on}(t)-\mu m_i^f+\mu m_i^b-\delta m_i^f \quad (4)$$

$$\frac{dE_i}{dt} = \sigma E_i\frac{p_i}{p_i+k}I_{on}(t), \quad (5)$$

where to indicate the onset of the time interval when VSG-specific immunity is active we have: $I_{on}(t) = 0$ if $t < \tau$ and $I_{on}(t) = 1$ if $t \geq \tau$. For the first variant, $i = 1$, the equations are the same, only there is no switching-to this variant, thus the term $+sv_{i-1}^{b}$ does not appear. In these equations $p_i = v_i^b + m_i^b + v_i^f + m_i^f$ describes the level of antigenic wave i over time in a typical infection. $V^f = \sum_i v_i^f$ and $V^b = \sum_i v_i^b$represent total level of slender cells in blood and fat respectively. $M^f = \sum_i m_i^f$ and $M^b = \sum_i v_i^b$represent total level of stumpy cells in blood and fat respectively. While by $P^b = \sum_i p_i$ and $P^f = \sum_i p_i$ we denote the total parasite levels in blood and fat. The proportion of stumpy cells at any time is given by the ratio $R^b = M^b/P^b$ and $R^f = M^f/P^f$, in the blood and fat compartments respectively.

This model captures several processes of infection dynamics. Cell division time can be different in blood and fat this is reflected in the population growth rates: rb, rf . The differentiation function (slender-to-stumpy) is density-dependent (1, 2), assumed linear with respect to total parasite load $V + M$ in each compartment, and is maximal when parasite densities reach K_b and K_f in blood and fat. Switching to new antigenic blocks (waves), indexed by i happens only in the blood, at probability s per cell division. Variant-specific immunity E_i is stimulated by all antigen presentation p_i across the blood and fat compartment, and killing rates of parasites per variant in blood and fat per unit of time are assumed equal. However, killing rates by immunity are different when considering slender and stumpy cells, in line with previous observations (3), denoted by d and d' respectively. The stimulation of host immune response by parasite density is a non-linear Hill function, denoting that as parasite density increases, immune stimulation saturates, with a half-maximal saturation constant given by k. This formulation is typical in models of antigenic variation (4), although here we don't consider the additional effects of a general variant-transcending immune response. The activation rate σ captures net rate of increase in VSG-specific immune response. Variant-specific immunity starts to respond at day τ post-inoculation, and only increases in the time-frame of infection that we model (no decay). It serves as a mechanism for new waves of parasite variants to have a relative advantage over existing variants, and thus for immune evasion via antigenic variation. We assume no cross-reactivity between immune responses.

All variants are assumed symmetric, in all their parameters. Stumpy cells have a fixed life-span, with denoting the death rate per unit of time. In this 'typical dynamics model', all parameters stay constant during the infection period, i.e. there is no deterioration in intrinsic growth rate, nor deterioration in immune response capacity of the host. We assume parasites can migrate between compartments at migration rate μ per cell per unit of time, equal for stumpy and slender cells. Even though there could be asymmetric migration for cells leaving the blood, and those leaving the adipose tissue, here we assume for simplicity that migration rates are symmetric in each direction.

Thus, even though, in principle there are multiple possibilities for trait variation in parasites across the blood and fat compartment, in this study we start by the simplest assumption, that most parameters are the same (Supplementary Table 5). Then, in particular, we focus only on the following nested hypotheses for plausible differences: growth rate differences (H1), and growth plus differentiation rate differences (H2). These are the two processes that have been mostly studied in the trypanosome literature. The null hypothesis is that there are no differences at all in parameters across compartments (H0). We fit these three models to the data and compare them statistically and also based on biological plausibility of inferred parameters.

Supplementary Table 5. Model parameters and interpretation.

Parameter	Interpretation	Units	Range
r_b, r_f	Growth rate of slender cells (blood, fat)	day^{-1}	$\in [1, 5]$
K_b, K_f	Differentiation parameters (in blood and fat)	cells/mg	$\log \in [8, 11]$
d	Minimal killing rates for slenders by immunity	day^{-1}	$\log \in [-10, -5]$
d'	Minimal killing rates for stumpies by immunity	day^{-1}	$\log \in [-10, -6]$
μ	Migration rate across compartments	day^{-1}	$\log \in [-5, 0]$
s	Switch probability per division to next antigenic wave	-	$\log \in [-12, -5]$
σ	Activation rate of anti-VSG immune response	day^{-1}	$\in [1, 5]$
k	Half-saturation constant for immune stimulation by antigen	cells/mg	$\log \in [5, 11]$
p_0	Initial density of parasites in blood ($t = 0$)	cells/mg	1 (fixed)
I_0	Initial level of VSG-specific response (reference)	-	1 (fixed)
n	Number of antigenic waves over 28 days	-	$n = 5$ (fixed)
δ	Mortality rate of stumpy cells	day^{-1}	$\delta = 0.5$ (fixed)
τ	Initial delay for immune response activation	days	$\tau = 4$ days (fixed)

Some key infection parameters are estimated from the data. Others model parameters are assumed fixed at biologically reasonable values, to avoid overfitting and identifiability problems. Infection starts with $2\text{x}10^3$ parasites injected intraperitoneally in each animal, which when scaled by the total volume of the blood result in approximately $p_o = 1$ cell/mg. The initial level of immune response is also set to a reference value of 1, such that any increase is relative (fold increase from onset), and thus d and d' can be seen to describe minimal rates of killing by VSG-specific immunity. The number of antigenic waves is set to 5, chosen to correspond broadly to the number of peaks expected over 28 days (5). Lifespan of stumpy cells is assumed 2 days, leading to a mortality rate of 0.5 per day (0.02 h^{-1}) (6). The initial delay for immune response activation is set to 4 days, in line with previous estimates of this parameter (6). (Note log above refers to natural logarithm).

Model fitting to data

The data consists in four time-series corresponding to 17 time points for measurements of parasite load in the blood and in the adipose tissue, as well as 14 time points for proportion of slender/stumpy cells in the blood and in the adipose tissue between day 1 and 28 post-inoculation. The time series for parasite loads were obtained as geometric means over replicates, while the stumpy proportions over time were obtained as arithmetic means over replicates. The model is developed to describe the average dynamics of a typical infection (Fig. S5), neglecting host-to-host variation and VSG-to-VSG variation in biological parameters. Our motivation was to fit the model to data to estimate the parameters driving the dynamics.

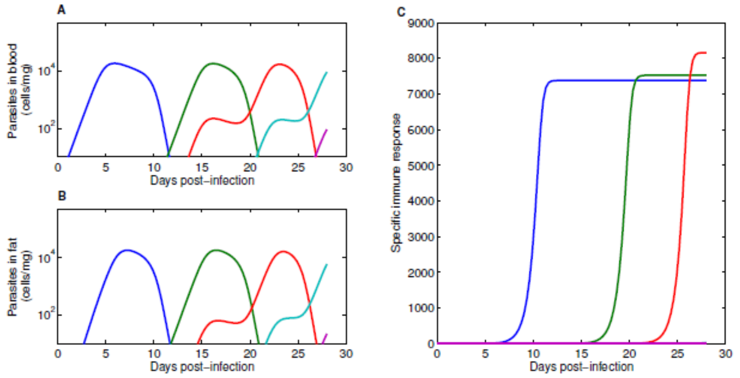

Supplementary Figure 5| A typical infection dynamics simulated by our model. A, Trypanosome dynamics across subsequent antigenic waves in the blood. **B,** Trypanosome dynamics in the adipose tissue. **C,** VSG-specific immune response growth (fold-increase) from its initial level 1. Although the model predicts more variables than we can fit with available data, we

use the aggregated total parasite densities in the blood, fat and stumpy proportions in the two compartments as model read-outs in our estimation procedure.

We consider different hypotheses for the biological differences between the blood and fat compartments where parasites can grow. First, we consider a null model where there are no differences (Model 1). This corresponds to estimating 8 parameters. Then we consider Model 2 with difference in growth rates of slender cells in the two compartments. This could be related to different opportunities for growth and nutrient utilization when parasites live extracellularly in the blood or in the adipose tissue ($rb \neq rf$). This model corresponds to estimating 9 parameters. Third, we consider a slightly more complex model: Model 3, where we allow that in addition to growth, differentiation may also happen at different rates in blood and adipose tissue. This is translated in two parameters: K_b and K_f , increasing the number of parameters to 10.

We constructed a likelihood function for the total error between the dynamic model and infection data, considering log parasite numbers and percentage stumpies, over all time points. Since we did not observe antigenic variant densities, but only total parasite numbers, the data can be expressed as a sequence of pairs $(t_k, \bar{P}_k^b)$ for total parasite numbers in blood, $(t_k, \bar{P}_k^f)$ for total parasite numbers in fat, and $(t_k, \bar{R}_k^b)$ for percentage stumpies in blood, $(\bar{R}_k^f)$ for percentage stumpies in fat, where the index k runs between 1 and T_{max}, over all sampled time points. For each sampled parameter combination θ, the mean squared error, between model and data, is given by:

$$E(\theta) = \frac{1}{N_k}\left(\sum_{t_k}[P^b(\theta,t_k) - \tilde{P}_k^b]^2 + \sum_{t_k}[P^f(\theta,t_k) - \tilde{P}_k^f]^2 + \sum_{t_k}[R^b(\theta,t_k) - \tilde{R}_k^b]^2 + \sum_{t_k}[R^f(\theta,t_k) - \tilde{R}_k^f]^2 \right)$$

Assuming a normal distribution for the error with mean 0 and variance ν^2, the likelihood of the data is:

$$P(data|\theta,\nu) \approx \frac{1}{\nu^2} e^{-E(\theta)/\nu^2}$$

For each parameter combination θ, we simulate the model trajectories using Equations 1-5 (obtaining P^b, P^f ,R^b, and R^f), and compare them with the data($\bar{P}^b$,$\bar{P}^f$, $\bar{R}^b$ and $\bar{R}^f$). This is done in an iterative manner using Markov Chain Monte Carlo sampling in Matlab. To implement MCMC parameter estimation in Matlab we used the adaptive MCMC mcmcstat package (7). We used uniform prior distributions for all parameters (in either log or linear scale), with ranges determined by preliminary numerical simulations and informed by the literature, as specified in Supplementary Table 5. Two MCMC chains were run until convergence to a stationary multivariate distribution for θ. Convergence was verified using the Gelman-Rubin statistic for all parameters (below 1.2) (8) as well as confirmed by visual inspection of the traces. This typically required 20.000 iterations in our case. After convergence, the Markov chains were run for a further 10.000 iterations to obtain the posterior distributions and their statistics. To compare different models, we considered the biological feasibility of the estimated parameter values, the Deviance Information Criterion (DIC), the likelihood ratio as the models are nested, as well as visual inspection of the 95% credible envelopes for the infection trajectories under each model.

Parameter estimation results

Supplementary Table 6. Model 1 (null model): no differences between blood and fat.

	r	$ln(d)$	$ln(d')$	$ln(K)$	$ln(k)$	$ln(\mu)$	σ	$ln(s)$
Mean	2.12	-6.44	-7.57	9.44	6.72	-4.45	1.38	-7.23
95%CI	1.85	-9.07	-9.75	8.14	5.36	-4.97	1.04	-9.20
	2.39	-5.06	-5.87	10.84	8.77	-3.47	1.83	-5.27

Supplementary Table 7. Model 2: growth rate differences between blood and fat.

	r_b	r_f	$ln(d)$	$ln(d^{'})$	$ln(K)$	$ln(k)$	$ln(\mu)$	σ	$ln(s)$
Mean	2.38	1.21	-5.73	-8.92	10.35	8.19	-2.23	1.68	-7.79
95%CI	2.10	1.01	-7.65	-9.96	8.81	6.90	-2.95	1.27	-9.05
	2.56	1.56	-5.02	-7.09	10.98	9.73	-1.66	2.21	-6.57

Supplementary Table 8. Model 3: Differentiation and growth rate differences between blood and fat.

	r_b	r_f	$ln(d)$	$ln(d^{'})$	$ln(K_b)$	$ln(K_f)$	$ln(k)$	$ln(\mu)$	σ	$ln(s)$
Mean	2.33	1.23	-5.84	-8.75	10.18	9.56	8.14	-2.76	1.67	-7.42
95%CI	2.14	1.01	-7.47	-9.92	8.30	8.10	7.07	-4.60	1.28	-8.47
	2.49	1.69	-5.04	-7.02	10.97	10.90	9.55	-0.18	2.21	-6.29

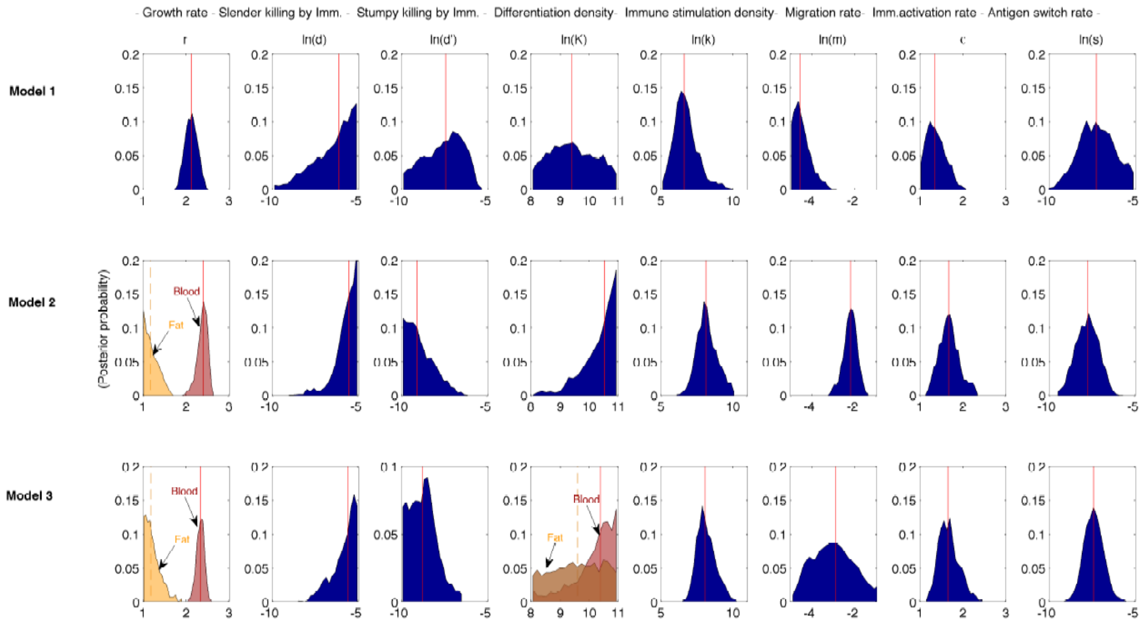

Supplementary Figure 6| Posterior distributions for model parameters for the 3 models considered. Model 1: null model with no differences between blood and fat. Model 2: with growth rate differences (yellow: r, red: rb). Model 3: with growth rate and differentiation rate differences (yellow: r_f, K_f , red: r_b, K_b). Red vertical lines denote the medians and red dashed lines, denote the medians for the parameters specific to the adipose tissue.

Model comparison

Our models converge on similar values for estimated parameters (Tables S6-S8 and Fig. S5), indicating they are also close biologically. The posterior distributions for the standard deviation of the error (v) under each model are shown in Supplementary Figure 6.

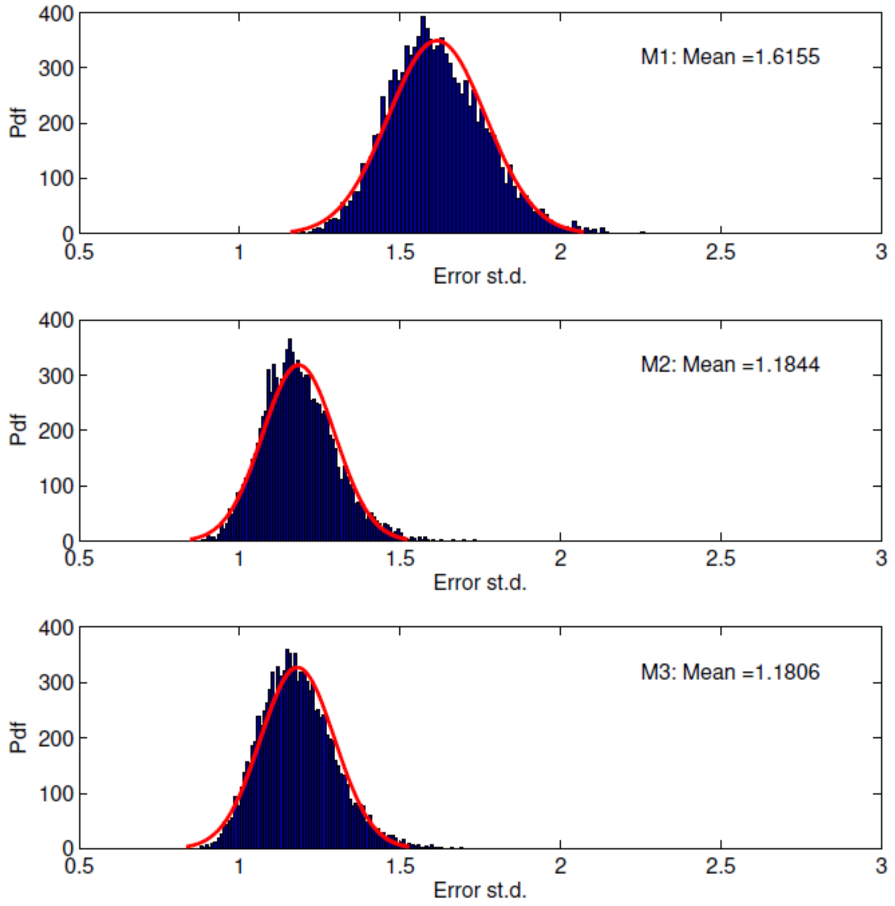

Supplementary Figure 7| Error posterior distributions. The likelihood function in our Bayesian estimation procedure is based on a normal distribution for the errors, with mean zero and standard deviation to be estimated from the data. The posterior distribution for the error std. (v) estimated via MCMC is shown here for each model (blue) with a normal curve fit superimposed (red).

It is clear that there is a significant improvement from Model 1 to Model 2, but no substantial improvement from adding an extra parameter in Model 3. This indicates Model 2 (growth rate differences model) as a better model compatible with the current data. This can also be verified with a marginal likelihood ratio test, where Model 2 is much more likely to apply than Model 1:

$$\frac{Prob(data|\text{Model 2})}{Prob(data|\text{Model 1})} = e^{33.8} = 5 \times 10^{14}, \text{ whereas } \frac{Prob(data|\text{Model 3})}{Prob(data|\text{Model 2})} = 1.25,$$

which means Model 2 is sufficient to explain the data, and no further complexity is needed. The Deviance Information Criterion (9), which in addition takes into account the uncertainty around parameters and number of parameters in each model (Supplementary Table 9), also indicates support for the model with parasite growth differences across in-host compartments.

Supplementary Table 9. Different model formulations to compare biological hypotheses on the same dataset.

Model	Parameter change	Biological assumption for difference in the blood-fat dynamics	Nr. parameters	DIC	Log-Likelihood
1	$r_b = r_f, K_b = K_f$	**No** differences in blood and fat	8	173.42	-65.26
2	$r_b \neq r_f$	**Growth rate** varies in blood and fat.	9	86.10	-31.44
3	$r_b \neq r_f$ $K_b \neq K_f$,	**Growth rate and differentiation rate** differ simultaneously.	10	85.13	-31.20

The models overall are able to capture accurately the global parasite dynamics across the blood and adipose tissue over 28 days (Fig. S8). Specifically, the model most supported by the data (Model 1), suggests a growth differential of about 50% between parasites growing in the different compartments, where parasites in the adipose tissue replicate less than those in the bloodstream.

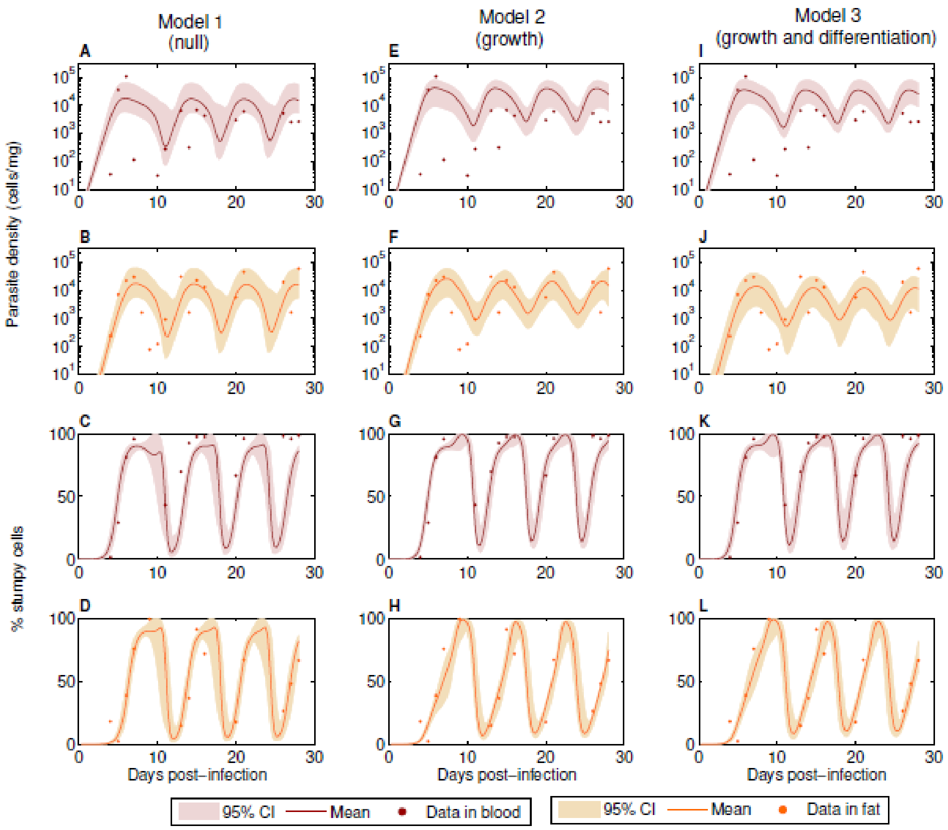

Supplementary Figure 8| Model fit and 95%CI for our models. A-D, (Model 1) **E-H,** (Model 2) and **I-L,** (Model 3). Scattered points indicate the data that were used to fit each model. The lines indicate the model prediction with mean parameters estimated via MCMC. The shaded region indicates the 95% credible envelope from 50 simulations with random parameters sampled from the posterior estimated via MCMC for each model.

The model therefore predicts more rapid increase in stumpy cell levels in the blood than in the adipose tissue, without substantial differences in total parasite loads (Fig. S9). This can be clearly seen from model fits to data, where the stumpy levels follow sharper acute oscillatory dynamics in the fat than in the blood.

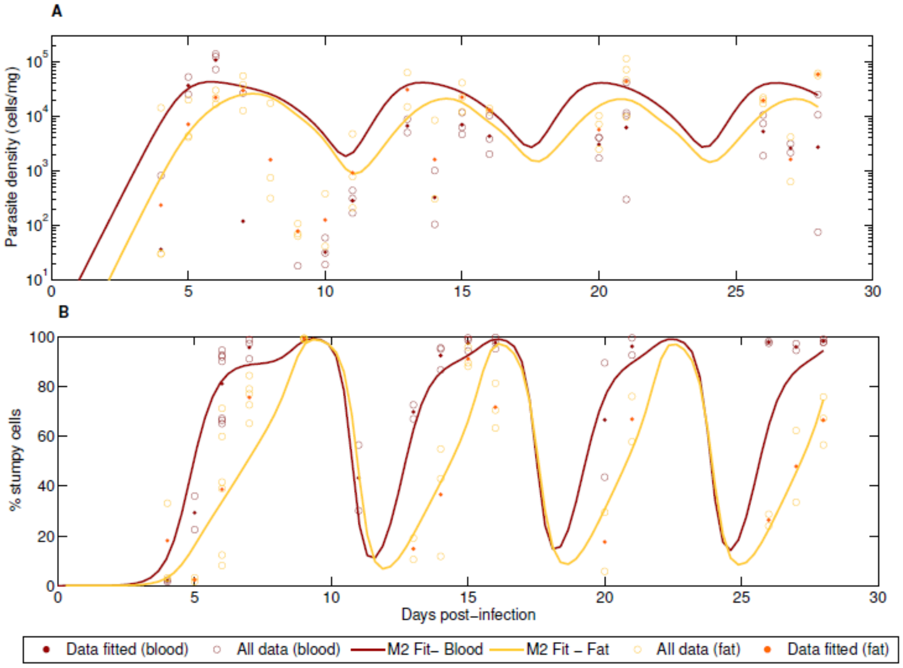

Supplementary Figure 9| Mean model fit for Model 2 vs. data, with blood and fat observations superimposed. A, Relative parasite levels in blood and fat. **B,** Stumpy proportions in blood and fat. Filled scattered points indicate the data used to fit the model, while empty circles indicate the full data (multiple observations for the same time point). The lines indicate the model prediction with mean parameters.

Model discussion

Because the model was built with the aim to capture global infection dynamics (typical infection averaged across many individual mice), there were simplifying assumptions regarding several aspects of the biology, known or expected from previous studies. We assumed no variation in switch rates, where it has been hypothesized that trypanosomes have an organized architecture of their antigenic repertoire, where switching rates may be discontinuous, such that within blocks switching happens at faster rate than between blocks (1, 2). We chose not to model such level of specificity because the number of parameters would increase significantly hampering their identifiability from global dynamics alone (i.e. no VSG variant dynamics). Similarly, we chose to model differentiation with one parameter only, assuming simple density-dependence, where other studies have suggested that cell heterogeneity in the gradual process from slender to stumpy, including intermediate forms may be important, and that SIF dynamics could play a significant role (10). Clearly, without such details, it is difficult to replicate all the intricate patterns observed in total parasite levels in the blood and fat, in their totality, especially the first trough of the parasitaemia in the blood, where our model is somewhat upwards biased (inevitably constrained by the match with temporally-linked stumpy proportions). However, since our primary aim was in probing differences across in-host compartments, we expect any bias in the model structure, should affect both compartments in a similar way and thus not contribute substantially to model comparison.
Homogeneity between variants in terms of growth potential, and time-homogeneous parameters were also two major assumptions. If these are changed (2, 11), they could give more degrees of freedom to fit any apparent outlier data points, or unique behavior of different peaks, but our power to estimate them confidently with the current dataset would be realistically limited. More detailed data, at the levels of single animal dynamics and antigenic variants should be more informative in the future to add such level of model complexity.

SI References

1. E Gjini, DT Haydon, JD Barry, and CA Cobbold. Critical interplay between parasite differentiation, host immunity, and antigenic variation in trypanosome infections. The American Naturalist, 176(4):424–439, 2010.
2. Katrina A Lythgoe, Liam J Morrison, Andrew F Read, and J David Barry. Parasite-intrinsic factors can explain ordered progression of trypanosome antigenic variation. Proceedings of the National Academy of Sciences, 104(19):8095–8100, 2007.
3. LML McLintock, CMR Turner, and K Vickerman. Comparison of the effects of immune killing mechanisms on *Trypanosoma brucei* parasites of slender and stumpy morphology. Parasite immunology, 15(8):475–480, 1993.
4. Rustom Antia, Martin A Nowak, and Roy M Anderson. Antigenic variation and the within-host dynamics of parasites. Proceedings of the National Academy of Sciences, 93(3):985–989, 1996.
5. Monica R Mugnier, George AM Cross, and F Nina Papavasiliou. The in vivo dynamics of antigenic variation in *Trypanosoma brucei*. Science, 347(6229):1470–1473, 2015.
6. Kevin M Tyler, Paul G Higgs, Keith R Matthews, and Keith Gull. Limitation of *Trypanosoma brucei* parasitaemia results from density-dependent parasite differentiation and parasite killing by the host immune response. Proceedings of the Royal Society of London. Series B: Biological Sciences, 268(1482):2235–2243, 2001.
7. Heikki Haario, Marko Laine, Antonietta Mira, and Eero Saksman. Dram: efficient adaptive mcmc. Statistics and computing, 16(4):339–354, 2006.
8. Walter R Gilks, Sylvia Richardson, and David Spiegelhalter. Markov chain Monte Carlo in practice. Chapman and Hall/CRC,1995.
9. David J Spiegelhalter, Nicola G Best, Bradley P Carlin, and Angelika Van Der Linde. Bayesian measures of model complexity and fit. Journal of the royal statistical society: Series b (statistical methodology), 64(4):583–639, 2002.
10. Paula MacGregor, Nicholas J Savill, Deborah Hall, and Keith R Matthews. Transmission stages dominate trypanosome within-host dynamics during chronic infections. Cell host & microbe, 9(4):310–318, 2011.
11. Dianbo Liu, Luca Albergante, TJ Newman, and David Horn. Faster growth with shorter antigens can explain a VSG hierarchy during African trypanosome infections: a feint attack by parasites. Scientific reports, 8(1):10922, 2018.
