# Supplementary material for: Slow growing behavior in African trypanosomes during adipose tissue colonization
Source: Nat Commun. 2022 Dec 8;13:7548. doi: 10.1038/s41467-022-34622-w (PMC9732351; doi:10.1038/s41467-022-34622-w)
Supplement: Supplementary file 3 — Description of Additional Supplementary Information [file 41467_2022_34622_MOESM3_ESM.docx]

**Description of Additional Supplementary Files for**

**Slow growing behavior in African trypanosomes during adipose tissue colonization**

Sandra Trindade, Mariana De Niz, Mariana Costa-Sequeira, Tiago Bizarra-Rebelo, Fábio Bento, Mario Dejung, Marta V. Narciso, Lara Lopez-Escobar, João Ferreira, Falk Butter, Frédéric Bringaud, Erida Gjini & Luisa M. Figueiredo.

Luisa M. Figueiredo, Erida Gjini

Email: lmf@medicina.ulisboa.pt and erida.gjini@tecnico.ulisboa.pt

**This PDF file includes:**

Legend for Supplementary Movie 1

Legend for Supplementary Data 1

**Other supplementary materials for this manuscript include the following:**

Supplementary Movie 1

Supplementary Data 1

Supplementary Data 2

Supplementary Data 3

Supplementary Movie 1. Intravital imaging of *T. brucei* parasites inside gonadal adipose tissue stromal fraction and vasculature. Lister 427 parasites from mice infected for 5 days. Vessels and tissue parenchyma are labelled with FITC-Dextran (yellow) and, kinetoplasts and nuclei with Hoechst (blue).

Supplementary Data 1. Proteomic analysis for Lister 427 BSFs and ATFs parasites. The mass spectrometry proteomics data have been deposited to the ProteomeXchange Consortium via the PRIDE partner repository with the dataset identifier PXD014958.

**Supplementary Data 2. Mathematical model fitting.** Code for mathematical modeling.

**Supplementary Data 3. Commercial Reagents List.** List of used commercial reagents and respective company and number.
