# Supplementary material for: Slow growing behavior in African trypanosomes during adipose tissue colonization
Source: Nat Commun. 2022 Dec 8;13:7548. doi: 10.1038/s41467-022-34622-w (PMC9732351; doi:10.1038/s41467-022-34622-w)
Supplement: Supplementary file 8 — Reporting Summary [file 41467_2022_34622_MOESM8_ESM.pdf]

nature portfolio

Corresponding author(s): Luisa M. Figueiredo and Erida Gjini

Last updated by author(s): Oct 21, 2022

Reporting Summary

Nature Portfolio wishes to improve the reproducibility of the work that we publish. This form provides structure for consistency and transparency in reporting. For further information on Nature Portfolio policies, see our Editorial Policies and the Editorial Policy Checklist.

Statistics

For all statistical analyses, confirm that the following items are present in the figure legend, table legend, main text, or Methods section.

n/a	Confirmed	
☐	☒	The exact sample size (*n*) for each experimental group/condition, given as a discrete number and unit of measurement
☐	☒	A statement on whether measurements were taken from distinct samples or whether the same sample was measured repeatedly
☐	☒	The statistical test(s) used AND whether they are one- or two-sided<br>*Only common tests should be described solely by name; describe more complex techniques in the Methods section.*
☐	☒	A description of all covariates tested
☐	☒	A description of any assumptions or corrections, such as tests of normality and adjustment for multiple comparisons
☐	☒	A full description of the statistical parameters including central tendency (e.g. means) or other basic estimates (e.g. regression coefficient) AND variation (e.g. standard deviation) or associated estimates of uncertainty (e.g. confidence intervals)
☐	☒	For null hypothesis testing, the test statistic (e.g. *F*, *t*, *r*) with confidence intervals, effect sizes, degrees of freedom and *P* value noted<br>*Give P values as exact values whenever suitable.*
☐	☒	For Bayesian analysis, information on the choice of priors and Markov chain Monte Carlo settings
☒	☐	For hierarchical and complex designs, identification of the appropriate level for tests and full reporting of outcomes
☒	☐	Estimates of effect sizes (e.g. Cohen's *d*, Pearson's *r*), indicating how they were calculated

Our web collection on statistics for biologists contains articles on many of the points above.

Software and code

Policy information about availability of computer code

Data collection	All technical aspects related to modeling and model-fitting to data are fully detailed in the Supplementary Information. Codes are available in the Supplementary Data 2.
Data analysis	Model fitting to data was performed under a Bayesian framework, using the adaptive Monte Carlo Markov chain mcmcstat package in Matlab.

For manuscripts utilizing custom algorithms or software that are central to the research but not yet described in published literature, software must be made available to editors and reviewers. We strongly encourage code deposition in a community repository (e.g. GitHub). See the Nature Portfolio guidelines for submitting code & software for further information.

Data

Policy information about availability of data

All manuscripts must include a data availability statement. This statement should provide the following information, where applicable:

- Accession codes, unique identifiers, or web links for publicly available datasets
- A description of any restrictions on data availability
- For clinical datasets or third party data, please ensure that the statement adheres to our policy

The number of independent replicates was pre-estimated by a power analysis performed on G*Power 3.1 software. The used data sets were anterior to this publication (2 different groups of infected mice).
TriTrypDB version 33 and UniProt protein databases were used in the proteomic analysis. The mass spectrometry proteomics data generated in this study have been

deposited to the ProteomeXchange Consortium via the PRIDE partner repository with the dataset identifier PXD014958. The proteomic analysis generated in this study is provided in the Supplementary Data 1.
Source Data are provided with this paper.
Figures 1 and 2 are linked to Table S1, Figure 2 is linked to Supplementary Informatio, Supplementary Data 2 and Tables S2 and S3 and Figure 3 is linked to Supplementary Data 1 and Table S4.

Human research participants

Policy information about studies involving human research participants and Sex and Gender in Research.

Reporting on sex and gender	N/A
Population characteristics	N/A
Recruitment	N/A
Ethics oversight	N/A

Note that full information on the approval of the study protocol must also be provided in the manuscript.

Field-specific reporting

Please select the one below that is the best fit for your research. If you are not sure, read the appropriate sections before making your selection.

[x] Life sciences [ ] Behavioural & social sciences [ ] Ecological, evolutionary & environmental sciences

For a reference copy of the document with all sections, see nature.com/documents/nr-reporting-summary-flat.pdf

Life sciences study design

All studies must disclose on these points even when the disclosure is negative.

Sample size	Most experiments were performed with 4 independent replicates. This number was pre-estimated by a power analysis performed on G*Power 3.1 software. The used data sets were anterior to this publication (2 different groups of infected mice). The defined number of replicates was considered for all the experiments also due to ethical usage of mice and in accordance to the 3R's policy; and only exceptionally (e.g. lack of statistical power for a specific tested condition) the number of replicates was higher than 4.
Data exclusions	In Figure 1A, for parasite densities, 6 blood samples (out of 46) and 6 adipose tissue samples (out of 46) were discarded due to sample deterioration. For the % of stumpy forms, 13 blood samples (out of 51) and 11 adipose tissue samples (out of 51) were discarded due to low counts or mice problems.<br>In Figure 4A-B and S2A, a total of 4 samples were discarded due to low counts, aberrant parasites or mice problems.<br>In Figure 4G and S2E, a total of 30 fields of view (out of 771) were discarded due to undetectable CTV signal or statistically tested outliers.<br>In Figure 5A, 2 experiments were excluded due to lack of healthy growth conditions for up to 1 week.
Replication	All experiments were performed more than once on an independent manner (biological replicates). All but one attempt at replication were successful. For cell proliferation in vitro, due to several technical reasons (e.g. sorting, dilution, growth conditions), 2 out of 4 performed experiments result in no healthy growth conditions for up to 1 week.
Randomization	Animals were allocated randomly into experimental groups. Exception for aparasitemic or very low parasitized infected animals, that were non randomly discarded.
Blinding	The investigators were not blinded to group allocation during data analysis. Blinding was not relevant to our study since most results depend on instrument acquisition followed by human analysis that was reviewed by a second independent investigator. For microscopy acquisitions, the investigators were also not blinded to group allocation. Blinding was also not relevant in this case since, the acquisitions were performed on arbitrarily chosen fields of view.

Reporting for specific materials, systems and methods

We require information from authors about some types of materials, experimental systems and methods used in many studies. Here, indicate whether each material, system or method listed is relevant to your study. If you are not sure if a list item applies to your research, read the appropriate section before selecting a response.

Materials & experimental systems

n/a	Involved in the study
x	Antibodies
	x Eukaryotic cell lines
x	Palaeontology and archaeology
	x Animals and other organisms
x	Clinical data
x	Dual use research of concern

Methods

n/a	Involved in the study
x	ChIP-seq
	x Flow cytometry
x	MRI-based neuroimaging

Eukaryotic cell lines

Policy information about cell lines and Sex and Gender in Research

Cell line source(s)	GFP::PAD1utr cell line from Christian Janzen laboratory (University of Wurzburg, Germany), Lister 427 from George Cross laboratory (The Rockefeller University, New York, USA)
Authentication	The cell lines were not authenticated
Mycoplasma contamination	The cell lines were not tested for Mycoplasma contamination
Commonly misidentified lines (See ICLAC register)	No commonly misidentified cell lines were used

Animals and other research organisms

Policy information about studies involving animals; ARRIVE guidelines recommended for reporting animal research, and Sex and Gender in Research

Laboratory animals	Mouse, C57BL/6J, Male, 8-13 week old
Wild animals	The study did not involve wild animals
Reporting on sex	Our findings apply only to male mice. Tissue tropism in trypanosomes was previously described for the adipose tissue of male mice, where gonadal adipose tissue has been studied in detail. This depot presents sexual dimorphism and therefore the biological phenotypes associated to it might differ between male and female mice.
Field-collected samples	The study did not involve samples collected from the field
Ethics oversight	Animal experiments were performed according to EU regulations and approved by the Órgão Responsável pelo Bem-estar Animal (ORBEA) of Instituto de Medicina Molecular and the competent authority Direcção Geral de Alimentação e Veterinária (license number: 018889 \2016).

Note that full information on the approval of the study protocol must also be provided in the manuscript.

Flow Cytometry

Plots

Confirm that:

- [x] The axis labels state the marker and fluorochrome used (e.g. CD4-FITC).
- [x] The axis scales are clearly visible. Include numbers along axes only for bottom left plot of group (a 'group' is an analysis of identical markers).
- [x] All plots are contour plots with outliers or pseudocolor plots.
- [x] A numerical value for number of cells or percentage (with statistics) is provided.

Methodology

Sample preparation	AnTat1.1 GFP::PAD1utr reporter parasites isolated from blood and adipose tissue were fixed, permeablized and stained with Propidium Iodide prior to flow cytometer analyses.<br>Lister 427 parasites from culture or isolated from blood and adipose tissue were fixed, permeabilized and stained with Click-iT™ HPG/DAPI/PI prior to flow cytometer analyses.<br>Lister 427 parasites from culture were stained with PKH26 or CellTrace™ Violet prior to flow cytometer analyses. Also, PKH26 or CellTrace™ Violet stained parasites isolated from blood and/or adipose tissue were analyzed and/or sorted by flow cytometry.
Instrument	BD LSRFortessa™, BD LSRFortessa™ X-20, BD FACSAria™ IIu, BD FACSAria™ III

Software	Flow cytometry data acquisitions were performed in FACSDiva 6.2 (Figure 1) and FACSDiva 8.0 (Figures 3, 4 and 5). Data analyses were performed in FlowJoTM 10.
Cell population abundance	Analyzed cell populations were within 1000s to 100000s events. Samples were either from isolated parasites or pure, with purity defined based on a gating strategy where a pure culture sample was used to gate for parasites followed (when applicable) by gating of DAPI/PI positive cells to exclude debris. Sorted cells purity was assessed based on the gates defined during the sorting process.
Gating strategy	SSC-A/FSC-A to determine parasites gate, FSC-W/FSC-A to exclude doublets, Count/Comp-PerCP-Cy5-5-A to determine PI+, Count/FITC-A- to determine the % of stumpy cells (threshold determined with culture GFP- and GFP+ cells).<br>SSC-A/FSC-A to determine parasites gate, FSC-W/FSC-A to exclude doublets, Count/Comp-Pacific Blue-A to determine DAPI+, Count/Comp- Texas Red-A- to determine HPG MFI (compensation performed with pure samples of double negative and DAPI + and HPG+ cells).<br>SSC-A/FSC-A to determine parasites gate, FSC-W/FSC-A to exclude doublets, Count/Texas Red-A- to determine PKH26 positive cells (threshold determined with culture PKH26- and PKH26+ cells).<br>SSC-A/FSC-A to determine parasites gate, FSC-W/FSC-A followed by SSC-W/SSC-A to exclude doublets, FSCA/Pacific Blue-A- to sort CTV positive/CTV negative cells (threshold determined with culture CTV- cells).

☒ Tick this box to confirm that a figure exemplifying the gating strategy is provided in the Supplementary Information.
